# Exosomal lncRNA UCA1 Derived From Pancreatic Stellate Cells Promotes Gemcitabine Resistance in Pancreatic Cancer *via* the SOCS3/EZH2 Axis

**DOI:** 10.3389/fonc.2021.671082

**Published:** 2021-11-19

**Authors:** Yuan Chi, He Xin, Zhaoyu Liu

**Affiliations:** Department of Radiology, Shengjing Hospital of China Medical University, Shenyang, China

**Keywords:** pancreatic cancer, hypoxia, pancreatic stellate cells, exosomes, lncRNA UCA1, gemcitabine resistance

## Abstract

**Objective:**

Pancreatic cancer is associated with poor prognosis and dismal survival rates. This study aims to investigate roles of lncRNA UCA1-loaded exosomes secreted by pancreatic stellate cells (PSCs) in Gemcitabine (Gem) resistance of pancreatic cancer under hypoxia, which involves the methylation of SOCS3 and EZH2 recruitment.

**Methods:**

The exosomes were isolated from PSCs and hypoxic PSCs (HPSCs), and co-cultured with pancreatic cancer cells transduced with manipulated lncRNA UCA1, EZH2, and SOCS3. The interaction among lncRNA UCA1, EZH2, and SOCS3 was characterized by RIP and ChIP assays. Next, MTT assay, flow cytometry and TUNEL staining and Transwell assay were used to detect cell viability, apoptosis, invasion, and migration. Gem-resistant pancreatic cancer cell line (GemMIA-R3) was established, which was applied in a mouse xenograft model of pancreatic cancer, with MTT assay to determine Gem sensitivity.

**Results:**

LncRNA UCA1 was highly expressed, while SOCS3 was poorly expressed in pancreatic cancer tissues. Hypoxia induced activation of PSCs and promoted release of exosomes. LncRNA UCA1 delivered by hypoxic PSC-derived exosomes (HPSC-EXO) regulated histone methylation level in SOCS3 gene region through recruitment of EZH2. *In vitro* and *in vivo* experimental results confirmed that lncRNA UCA1-loaded HPSC-EXO promoted malignant phenotypes, inhibited apoptosis, and promoted Gem resistance of pancreatic cancer cells as well as tumorigenesis in mice.

**Conclusion:**

Under hypoxic conditions, exosomes secreted by hypoxia-induced PSCs deliver lncRNA UCA1 into pancreatic cancer cells, where lncRNA UCA1 recruits EZH2 and regulates histone methylation level in SOCS3 gene region, thereby augmenting pancreatic cancer resistance to Gem.

## Introduction

Pancreatic cancer has a poor prognosis and accounts for about 3% of new cancer cases each year (1). Pancreatic stellate cells (PSCs) are the main cell components of pancreatic cancer, which produce large number of extracellular matrix components (2). Increasing evidence has shown that hypoxia is a key microenvironment factor to promote the activation of PSCs (3). In pancreatic cancer, the accumulation of extracellular matrix leads to vascular collapse, impaired drug delivery and acquired chemoresistance, which may be induced by activated PSCs (4). Various factors secreted by stromal cells have recently been reported to promote gemcitabine (Gem) resistance (5). Gem is a nucleoside analogue, which has been the mainstay of pancreatic cancer chemotherapy for a long time. However, the clinical efficacy of Gem is limited due to its limited cellular uptake and impaired intracellular activation, resulting in low overall efficacy (6). Therefore, it is necessary to understand the biological characteristics of pancreatic cancer.

The hypoxic microenvironment, an important feature of solid tumors, promotes tumor cells to release exosomes (7). It is well known that exosomes secreted by multiple cells can mediate intracellular information transmission through delivering protein, messenger RNAs (mRNAs), microRNAs (miRNAs), and long noncoding RNAs (lncRNAs) between cells (8, 9). Interestingly, in order to adapt to the hypoxic microenvironment, many lncRNAs are highly expressed in tumor cells, encapsulated in exosomes, and transported to the corresponding receptor cells, thus conferring important roles in intercellular communication (7). For example, tumor cells can produce lncRNA UCA1-rich exosomes, which are transported to normoxic cells to promote tumor cell malignant phenotypes under hypoxia (10). Further, hypoxic exosomal lncRNA UCA1 has been found to promote drug resistance, angiogenesis, and tumor growth in pancreatic cancer (7, 11).

Moreover, lncRNA UCA1 is physically linked to the enhancer of zeste homolog 2 (EZH2), which inhibits p27Kip1 through histone methylation (H3K27me3) in p27Kip1 promoter, thus promoting tumorigenesis in nude mice in hepatocellular carcinoma (12). In addition, CTCF dependent recruitment of EZH2 to the SOCS3 gene promoter is likely to participate in the epigenetic silencing of SOCS3 and in regulating its gene expression in cancer development (13). SOCS3 overexpression has been observed to suppress malignant behaviors and Gem resistance of pancreatic cancer cells (14). Therefore, the present study set out to elucidate the roles of lncRNA UCA1 loaded in PSC-derived exosomes in Gem resistance of pancreatic cancer under hypoxia, and to identify the downstream mechanisms involving histone methylation in SOCS3 gene region and EZH2 recruitment.

## Methods

### Ethics Statement

The study was conducted under the approval of the Ethics Committee of Shengjing Hospital of China Medical University. All participating patients signed informed consent documentation. Animal experiments were approved by the Animal Ethics Committee of Shengjing Hospital of China Medical University. Extensive efforts were made to ensure minimal suffering of the included animals.

### Sample Collection

From November 2018 to October 2019, the pancreatic cancer tissues and adjacent normal tissues (5 cm from the edge of cancer tissue) of 35 patients with pancreatic cancer (aged 38-62 years old with a mean age of 52.8 ± 5.9 years; 23 males and 12 females) were collected in Shengjing Hospital of China Medical University. According to the staging classification proposed by Hermreck et al. (15), patients were clinically staged, with 10 cases of well-differentiated, 14 cases of intermediate stage, and 11 cases of poorly differentiated. All pathological specimens were confirmed in the pathology department, and patients treated before surgery and patients without surgery were excluded. Tissue sections were frozen in liquid nitrogen and stored at -80°C.

### Bioinformatics Analysis

Through the Gene Expression Omnibus (GEO) database (https://www.ncbi.nlm.nih.gov/geo/), the pancreatic cancer related GSE32676 and GSE16515 datasets were downloaded with GPL570 and GPL10787 as the carrier platforms. GSE32676 dataset contains 7 normal samples and 25 pancreatic cancer samples, and GSE16515 dataset contains 16 normal samples and 36 pancreatic cancer samples. Differential expression analysis was performed using R language “limma” package (http://www.bioconductor.org/packages/release/bioc/html/limma.html), with |logFC| > 1 and p value < 0.05 set as the screening criteria to screen out the differentially expressed genes in pancreatic cancer and adjacent normal tissues.

### Cell Culture, Grouping, Transfection and Hypoxia Culture

Human PSC cell line was obtained from the Mingzhou Biological Technology Co., Ltd. (Ningbo, China), and pancreatic cancer cell line PANC-1 and HEK-293T cells were purchased from Wuhan Procell Life Science & Technology Co., Ltd. (Wuhan, China). All cells were cultured in Dulbecco’s modified Eagle’s medium (DMEM; Hyclone, Beijing, China) containing 10% fetal bovine serum (FBS; Gibco, Invitrogen), 100 mg/mL penicillin and 100 mg/mL streptomycin at 37°C in 5% CO_2_ and 95% saturated humidity. When the cell confluence was about 80%, the cells were passaged. HPSCs were cultured under the same conditions as normal pancreatic epithelial cells and pancreatic cancer cell lines. Hypoxia was induced by 12-h incubation of PSCs in a nitrogen-filled incubator (94% N_2_, 5% CO_2_, 1% O_2_) (Thermo Fisher Scientific Inc., Waltham, MA), and obtained hypoxic PSCs were hereinafter referred as HPSCs. Three biological replicates were set for each group.

Before transfection, the cells in logarithmic phase were seeded into 6-well plates at a density of 1 × 10^5^ cells/well. The cells were transfected with lentiviral vectors (Invitrogen) according to the instructions of Lipofectamine 2000 transfection reagent (11668-019, Invitrogen) (**Supplementary Table 1**).

### Extraction and Identification of Exosomes

Exosomes in human PSCs and HPSCs (PSCs-EXO, HPSC-EXO) were isolated and extracted by total exosome kit (Thermo Fisher Scientific). The specific operation was as follows: the culture supernatant was collected, centrifuged at 4°C, 2000 g for 30 min, and the cells and debris were removed. The supernatant (4000 g) was centrifuged at room temperature for 15 min to enrich the exosomes, and then mixed with 0.5-fold volume of total exosomes separation reagent. The mixture was incubated overnight at 4°C and then centrifuged at 4°C for 60 min the next day. The precipitated exosomes were isolated and resuspended with PBS or stored at -80°C.

Identification: BCA protein detection kit (product No. 23228; Thermo Fisher Scientific) was used to determine the protein concentration in PSCs-EXO and HPSC-EXO. In addition, the protein levels of CD9 (Abcam, ab92726, 1:2000) and CD63 (Abcam, ab59479, 1:1000) were detected by Western blot. The exosome morphology and size were observed by a transmission electron microscope (TEM; Morgagni 268D, Philips, Holland). The size and size distribution of the PSCs-EXO and HPSC-EXO were detected by Malvern NanoSight NS300.

TEM observation: 20 μL fresh samples of PSCs-EXO and HPSC-EXO were loaded into the carbon coated copper electron microscope for 2 min, and then negatively stained with phosphotungstic acid solution (12501-23-4, Sigma-Aldrich) for 5 min. The EVs were then washed three times with PBS to remove the excess phosphotungstic acid solution, and then kept semi dry with filter paper. The image was observed at 80KV in Hitachi H-7650 TEM (Hitachi High-Tech, Tokyo, Japan).

Nanosight nanoparticle size analysis: 20 μg PSCs-EXO and HPSC-EXO was dissolved in 1mL PBS for 1 min, and the uniform distribution of PSCs-EXO and HPSC-EXO was maintained. Then, the particle size distribution of PSCs-EXO and HPSC-EXO was directly observed and measured by nanosight nanoparticle tracking analyzer (NTA, Malvern Instruments, Malvern, UK).

### MTT Assay

MTT method (Dojindo, JPN) was used to analyze cell proliferation. PANC-1 cells in logarithmic growth phase were seeded into 96-well culture plate at a density of 1 × 10^3^ cells/well. The cells were then transfected and placed in a cell incubator with 3 repeated wells in each group. After the cells were cultured for 96 h, each well was added with 100 µL of 0.5 mg/mL MTT buffer and cultured in an incubator for 2 h. A plate reader (Bio-Rad Laboratories, Hercules, CA) was then used to measure the OD value of each well at 490 nm.

For the gemcitabine response of PANC-1 cells, PANC-1 cells were pretreated according to the instructions and seeded in a 96-well plate at a density of 3000 cells per well, with 3 replicate wells in each group. After 12 h, the cells were incubated with 10 nM Gem (G6423, Sigma-Aldrich) for 1, 2, 3, 4, and 5 days. The cells were then incubated with 100 μL of 0.5 mg/mL MTT buffer for 2 h. The OD value of each well at 490 nm was measured using a multi-well plate reader (Bio-Rad).

### Flow Cytometry

After 48 h of transfection, the cells were digested with 0.25% trypsin (without EDTA) and centrifuged. According to the instructions of Annexin-V-FITC cell apoptosis detection kit (556547, Shanghai shuojia Biotechnology Co., Ltd., Shanghai, China), the cells were resuspended in 500 μL Binding Buffer and incubated with 5 μL Annexin V-FITC at room temperature for 15 min, followed by incubation with 10 μL PI on ice for 5 min in the dark. Next, 1 × 10^6^ cells were resuspended in 100 μL of dye solution. After incubation at room temperature for 15 min, 1 mL of HEPES buffer solution was added. The fluorescence of FITC and PI was detected at 515 nm and 620 nm respectively.

### qRT-PCR

TRIzol reagent (Thermo Fisher Scientific) was used to extract total RNA from tissues and cells. Then 1 μg total RNA was reversely transcribed into cDNA using reverse transcription kit PrimeScript RT reagent Kit with gDNA Eraser (TaKaRa, Japan). qRT-PCR was performed using SYBR Premix Ex Taq II (TaKaRa) in an ABI 7500 qPCR instrument (Thermo Fisher Scientific) with glyceraldehyde-3-phosphate dehydrogenase (GAPDH) serving as internal reference. The designed primers are shown in **Supplementary Table 2**. The relative transcription expression of target genes was calculated *via* relative quantitative method (2^-ΔΔCT^ method).

### Western Blot

The total protein was extracted by radioimmunoprecipitation assay (RIPA) lysate (Beijing Solarbio Science & Technology Co., Ltd., Beijing, China), and the protein concentration was determined by BCA kit (GBCBIO Technologies, Guangdong, China). The proteins were separated by polyacrylamide gel electrophoresis and transferred to polyvinylidene fluoride membranes (Millipore Corp., Billerica, MA), and 5% BSA was used to seal the proteins at room temperature for 1 h. Diluted primary rabbit antibodies (**Supplementary Table 3**) were used to incubate the proteins overnight at 4°C with GAPDH used as internal reference. The next day, secondary antibody goat anti-rabbit IgG antibody was added to the membranes and incubated for 2 h. The membranes were then reacted with enhanced chemiluminescence solution, developed in Image Quant LAS 4000C gel imager (GE Healthcare, Little Chalfont, UK), and analyzed using Quantity One v4.6.2 software.

### Fluorescent Labeling and Transfer of Exosomes

Cells at the logarithmic growth phase were collected for this experiment. The isolated exosomes were incubated with lipophilic dye DiO (Thermo Fisher Scientific) at a working concentration of 10 μM at 37°C for 20 min, and centrifuged at 12000 ×g and 4°C for 70 min. The PANC-1 cells (3 × 10^4^ cells per well) were incubated with DiO-labeled exosomes (25 μg/mL) for 24 h.

In order to confirm the transfer of lncRNA UCA1, HPSCs were transfected with Cy3-labeled lncRNA UCA1. HPSCs and PANC-1 cells expressing Cy3-lncRNA UCA1 were co-cultured for 48 h in a 24-well Transwell chamber. Next, PANC-1 cells were prepared as described above for immunofluorescence. In addition, the cytoskeleton of PANC-1 cells was stained with TRITC-Phalloidin (100 nM, 40734ES75, Yeasen Company, Shanghai, China) or FITC Phalloidin (100 nM, 40734ES80, Yeasen). Finally, a confocal microscope was used to determine the internalization of exosomes or exosomal lncRNA UCA1.

### Transwell Assay for Cell Invasion and Migration

Transwell chamber (pore size of 8 mm; Corning Incorporated, Corning, NY) in 24-well plates was used for this experiment. For the invasion experiment, 100 μL of Matrigel (BD Biosciences) was placed in each chamber before the experiment and solidified at 37°C for 2 h. The cells were digested, washed twice with PBS, and resuspended in serum-free DMEM/F12 medium to a density of 3 × 10^5^ cells/mL. Each group had 3 chambers, 200 μL cell suspension for each. Thereafter, 700 μL 10% DMEM was added to the lower chamber and incubated in a 37°C incubator with 5% CO_2_. After 48 h, the cells were fixed with 4% paraformaldehyde for 30 min, and stained with 0.05% crystal violet (G1062, Solarbio) for 5 min. The invaded cells were counted and photographed under an Olympus IX 71 microscope (Olympus, Tokyo, Japan).

### TUNEL Staining

*In situ* apoptosis detection kit (KeyGen BioTech Ltd, Jiangsu, China) was used to detect genomic DNA breakage during cell apoptosis. The sample was incubated with 100 μL of proteinase K solution at 37°C for 30 min. Next, 50 μL of TdT enzyme solution was added to the cell sample and incubated at 37°C in the dark for 60 min. Following this, the cells were incubated with 5 μL of streptavidin fluorescein solution and 45 μL of labeling buffer in the dark at 37°C for 30 min and stained with DAPI (Sigma-Aldrich) before observation under a fluorescence microscope.

### Chromatin Immunoprecipitation

Cells (5 × 10^6^) were fixed in 1% formaldehyde for 30 min at room temperature and subjected to ultrasonic treatment to produce 150-900 bp chromatin fragments. The chromatin was then immunoprecipitated with antibodies to EZH2, H3K27me3 or normal rabbit IgG (serving as NC). qRT-PCR was used to quantify the purified chromatin. The signals obtained from the ChIP assay were divided by the signals obtained from an Input sample. In this assay, 1% of starting chromatin served as the Input, and a dilution factor of 100 or 6.644 cycles (log2 of 100) was subtracted from the Ct value of the diluted Input. The primer is shown in **Supplementary Table 4**.

### RNA-Binding Protein Immunoprecipitation Assay

TRIzol reagent (Thermo Fisher Scientific) was used to extract total RNA from PSC cells. mRNA in the total RNA was isolated and purified by PolyATtract^®^ mRNA Isolation Systems (A-Z5300, A&D Technology Corporation, Beijing, China). Anti-EZH2 antibody (36-6300, Thermo Fisher Scientific) or anti-IgG antibody (ab124055, Abcam) was added into IP buffer (20-mM Tris pH 7.5, 140-mM NaCl, 1% NP-40, 2-mM EDTA) to incubate with protein A/g magnetic beads for 1 h for binding. The purified mRNA and magnetic bead-antibody complex were added into IP buffer supplemented with ribonuclease inhibitor and protease inhibitor, and were kept overnight at 4°C. The RNA was eluted with eluent buffer. After extraction and purification by phenol chloroform, lncRNA UCA1 was analyzed by qRT-PCR.

### Establishment of Gem-Resistant Cell Line

A Gem-resistant cell line was induced in MIA PaCa-2 pancreatic cancer cell line by increasing the concentration of Gem (0.25 µM, 1 µM or 3 µM) in the medium. It took about a week for cells to acquire resistance and resume cell proliferation with each increase of Gem concentration. The whole process took several months to reach the most resistant cell line GemMIA-R3 with the highest Gem concentration (3.0 μm). At each point, the cells were amplified, cryopreserved, and maintained at a concentration in a medium containing 3.0 μM. Prior to the use of specific anti-3.0 μM cell line in any experiment, Gem selection pressure was relieved for 7 days to avoid Gem interference. The concentration response curve and cell growth inhibition data are shown in **Supplementary Figure 1**.

### Pancreatic Cancer Xenografting Mice Model

Four-to-six-week-old NOD/SCID mice were raised under specific pathogen-free conditions at 26 - 28°C and 50 - 65% humidity. The grouping of mice was consistent with the cell grouping (**Supplementary Table 1**). Cancer cells in the pancreas were counted and resuspended in PBS at a final concentration of 2 × 10^7^ cells/mL. In addition, trypan blue exclusion method determined that 95% of the cells were viable before injection. The cells were then subcutaneously injected into the NOD/SCID mice. At the same time, 20 μg exosomes were injected into the mice *via* tail vein every three days. Two weeks after implantation, the mice were treated with 80 mg/kg Gem (G6423; Sigma-Aldrich) *via* intraperitoneal injection, three times a week for five weeks. The tumor volume was measured with Vernier calipers every week and calculated using the following formula: tumor volume = 1/2 × a × b^2^ (a is long diameter and b is short diameter). After 30 days, the mice were euthanized, and the tumor tissues were collected and weighed. Meanwhile, paraffin embedded tissues were prepared for subsequent experimental analysis.

### Immunohistochemistry

Tumor tissues were fixed in 10% neutral formalin, embedded in paraffin and cut into 5-μm-thick sections. Immunohistochemistry was performed on a Ventana Discovery Ultra automatic dyeing machine (Ventana Medical Systems, Roche Diagnostics, Indianapolis). The cover glass was loaded on the machine whereupon the sections were dewaxed and rehydrated, followed by endogenous peroxidase activity elimination and antigen retrieval. The sections were incubated with anti-Ki67 antibody (ab16667, Abcam), developed with DISCOVERY ChromoMap DAB Kit and counterstained with hematoxylin (Ventana). The images were collected with a DMi8 Leica microscope and analyzed with Image-Pro Premier software.

### Statistical Analysis

Statistical analysis was conducted using the SPSS 21.0 software (IBM, Armonk, New York). Measurement data were expressed as mean ± standard deviation. The data between two groups were compared using the independent sample *t* test. Paired *t* test was used for comparison between cancer tissues and adjacent normal tissues. The data comparison among multiple groups was performed using one-way analysis of variance (ANOVA), followed by the Tukey’s *post hoc* test. The data at different time points were compared by repeated measures ANOVA, followed by Bonferroni’s *post hoc* test. Pearson’s correlation coefficient was used for correlation analysis. A value of *p* < 0.05 was indicative of a statistically significant difference.

## Results

### Hypoxia Induces Activation of PSCs and Promotes the Release of Exosomes

**Figure 1:**
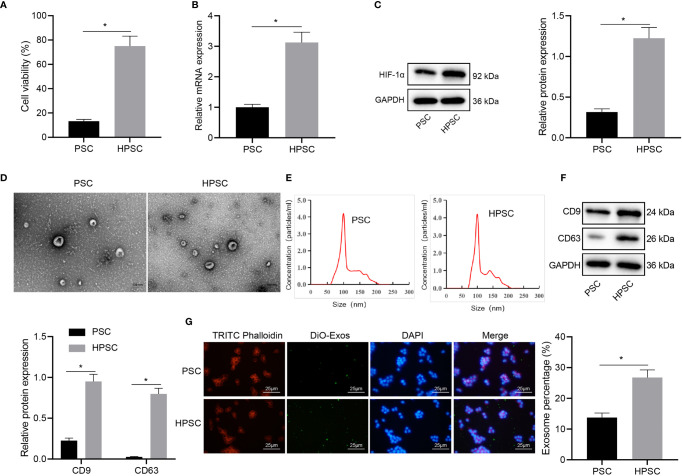
Hypoxia induces PSC activation and exosome release. **(A)**, Viability of PSCs and HPSCs was detected by MTT assay. **(B)**, Expression of HIF-1 in PSCs and HPSCs was detected by qRT-PCR. **(C)**, Expression of HIF-1 in PSCs and HPSCs was detected by Western blot. **(D)**, Morphology of exosomes isolated from PSCs and HPSCs (PSC-EXO and HPSC-EXO) observed under a TEM. **(E)**, NTA of the size distribution of PSC-EXO and HPSC-EXO. **(F)**, Expression of exosome marker protein in PSC-EXO and HPSC-EXO detected by Western blot. **(G)**, Immunofluorescence analysis of the uptake of DiO-labbeled PSC-EXO and HPSC-EXO by PANC-1 cells. *p < 0.05. Measurement data were expressed as mean ± standard deviation. Independent sample *t* test was applied for the comparison of data of two groups. The cell experiment was repeated three times.

Evidence exists reporting that hypoxia can induce the activation of PSCs (3), but the mechanism is unclear. In order to understand the effect of hypoxia on the activation of PSCs, we assayed the activities of PSCs cultured in normal environment and HPSCs cultured in hypoxia. The MTT results (**Figure 1A**) showed that compared with PSCs, hypoxia induced higher viability in HPSCs. Consistently, the expression of hypoxia inducible factor-1α (HIF-1α), as measured by qRT-PCR and Western blot, was up-regulated in HPSCs relative to PSCs (**Figures 1B, C**), which was consistent with the published literature (3, 16). The isolated PSC-EXO and HPSC-EXO were observed by a TEM, and the typical double-layer membrane and cup-shaped structure of exosomes could be observed (**Figure 1D**). Meanwhile, NTA (**Figure 1E**) showed irregular Brownian motion and an average diameter of 96.1 nm and 106.5 nm for the size distribution of PSC-EXO and HPSC-EXO. In addition, Western blot data (**Figure 1F**) showed that exosome marker proteins (CD9 and CD63) were significantly expressed in exosomes.

To determine whether the PSC-EXO and HPSC-EXO were absorbed by pancreatic cancer cells, exosomes were labeled with DiO and cytoskeleton with TRITC Phalloidin, followed by co-culture with PANC-1 cells for 24 h. Confocal microscopic observation results showed that DiO-labeled exosomes were internalized by PANC-1 cells (**Figure 1G**). The above results demonstrate the successful isolation of the exosomes and that hypoxia could induce PSC activation and exosome release.

### HPSC-EXO Promotes Gem Resistance and Tumorigenesis of Pancreatic Cancer Cells

**Figure 2:**
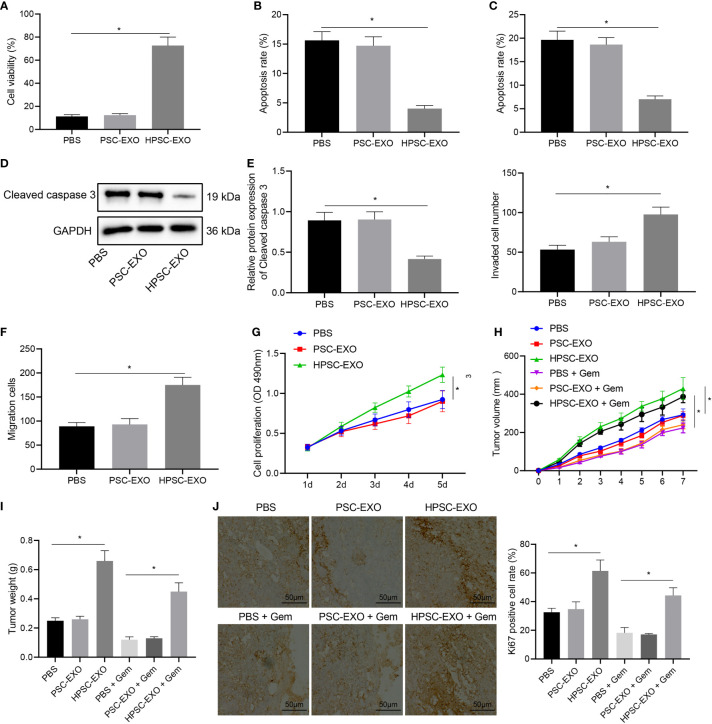
HPSC-EXO facilitates Gem resistance and tumorigenesis of pancreatic cancer cells. **(A)**, Viability of PANC-1 cells treated with PSC-EXO and HPSC-EXO was detected by MTT assay. **(B)**, Flow cytometric analysis of apoptosis of PANC-1 cells treated with PSC-EXO and HPSC-EXO. **(C)**, Apoptosis of PANC-1 cells treated with PSC-EXO and HPSC-EXO was detected by TUNEL staining. **(D)**, Cleaved caspase 3 protein expression in PANC-1 cells treated with PSC-EXO and HPSC-EXO was detected by Western blot. **(E)**, Migration of PANC-1 cells treated with PSC-EXO and HPSC-EXO was detected by Transwell assay. **(F)**, Invasion of PANC-1 cells treated with PSC-EXO and HPSC-EXO was detected by Transwell assay. **(G)**, Gem sensitivity of PANC-1 cells treated with PSC-EXO and HPSC-EXO was detected by MTT assay. **(H)**, Tumor volume of mice treated with PSC-EXO and HPSC-EXO after injection of Gem. **(I)**, Tumor weight of mice treated with PSC-EXO and HPSC-EXO after injection of Gem. **(J)**, The positive expression rate of Ki67 in tumor tissues of mice treated with PSC-EXO and HPSC-EXO after injection of Gem was detected by immunohistochemistry. *p < 0.05. Measurement data were expressed as mean ± standard deviation. The data comparison among multiple groups was performed using one-way ANOVA, followed by the Tukey’s *post hoc* test. The data at different time points were compared by repeated measures ANOVA, followed by Bonferroni’s *post hoc* test. The cell experiment was repeated three times. n = 8 for mice following each treatment.

PANC-1 cells were treated with HPSC-EXO or PSC-EXO to determine the possible effect of exosomes on the biological functions of PANC-1 cells. MTT assay results (**Figure 2A**) found that the cell viability in the presence of HPSC-EXO was the highest. Meanwhile, the results of flow cytometry (**Figure 2B**) and TUNEL staining (**Figure 2C**) showed that PSC-EXO treatment had no effect on cell apoptosis but HPSC-EXO treatment inhibited PANC-1 cell apoptosis. In addition, Western blot results (**Figure 2D**) also showed that the expression of cleaved caspase 3 was diminished only in the presence of HPSC-EXO, as compared with PBS treatment. The results of Transwell assay revealed that only HPSC-EXO treatment enhanced the migration and invasion of PANC-1 cells (**Figures 2E, F**).

Next, we tested the effect of HPSC-EXO on the gemcitabine resistance of PANC-1 cells. PANC-1 cells were incubated with Gem (10 μM), the proliferation of which was determined by MTT assay. The results suggested that HPSC-EXO promoted the resistance of PANC-1 cells to Gem, while PSC-EXO showed no influence on the cell resistance (**Figure 2G**).

Next, we shifted to further verify the effect of HPSC-EXO on pancreatic cancer cells *in vivo*. As shown in **Figures 2H–J**, HPSC-EXO treatment led to higher tumor volume and weight of mice and positive rate of the proliferation marker Ki67 in the tumor tissues than PBS treatment while PSC-EXO treatment showed no influence. In addition, HPSC-EXO treatment also significantly alleviated the inhibition of Gem on the tumor growth and Ki67 expression. These results suggest that HPSC-EXO can accelerate the tumorigenesis of pancreatic cancer cells and Gem resistance.

### LncRNA UCA1-Loaded HPSC-EXO Promote the Malignant Phenotypes and Gem Resistance of Pancreatic Cancer Cells

**Figure 3:**
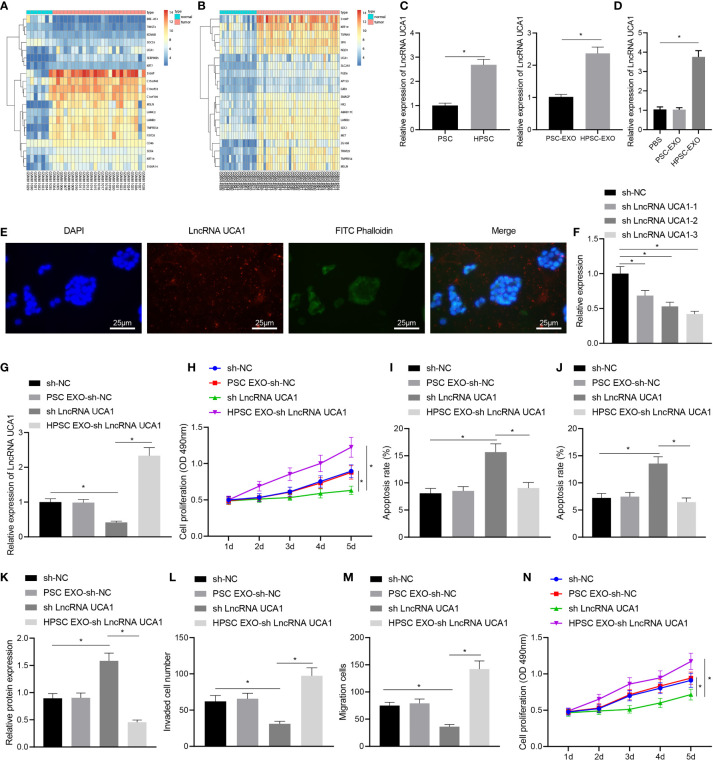
HPSC-EXO transfer lncRNA UCA1 to pancreatic cancer cells, where lncRNA UCA1 stimulates the malignant phenotypes of pancreatic cancer cells and their resistance to Gem. **(A)**, A heatmap of the expression of the first 10 differentially expressed genes in pancreatic cancer samples in the GSE32676 dataset. **(B)**, A heatmap of the expression of the first 10 differentially expressed genes in pancreatic cancer samples in the GSE16515 dataset. **(C)**, Expression of lncRNA UCA1 in PSCs and HPSCs as well as their derived exosomes was detected by qRT-PCR. **(D)**, Expression of lncRNA UCA1 in PANC-1 cells treated with PSC-EXO and HPSC-EXO was detected by qRT-PCR. **(E)**, Co-localization of lncRNA UCA1 and exosomes in PANC-1 cells was observed by a fluorescence microscope. **(F)**, Transfection efficiency of three lncRNA UCA1 shRNA sequences in PANC-1 cells was detected by qRT-PCR. **(G)**, Expression of lncRNA UCA1 in the PANC-1 cells treated with sh-lncRNA UCA1 or combined with HPSC-EXO was detected by qRT-PCR. **(H)**, Viability of PANC-1 cells treated with sh-lncRNA UCA1 or combined with HPSC-EXO was detected by MTT assay. **(I)**, Apoptosis of PANC-1 cells treated with sh-lncRNA UCA1 or combined with HPSC-EXO was detected by flow cytometry. **(J)**, Apoptosis of PANC-1 cells treated with sh-lncRNA UCA1 or combined with HPSC-EXO was detected by TUNEL staining. **(K)**, Cleaved caspase 3 protein expression in the PANC-1 cells treated with sh-lncRNA UCA1 or combined with HPSC-EXO was detected by Western blot. **(L)**, Migration of PANC-1 cells treated with sh-lncRNA UCA1 or combined with HPSC-EXO was detected by Transwell assay. **(M)**, Invasion of PANC-1 cells treated with sh-lncRNA UCA1 or combined with HPSC-EXO was detected by Transwell assay. **(N)**, Gem sensitivity of PANC-1 cells treated with sh-lncRNA UCA1 or combined with HPSC-EXO was detected by MTT assay. *p < 0.05. Measurement data were expressed as mean ± standard deviation. The data between two groups were compared using the independent sample *t* test. The data comparison among multiple groups was performed using one-way ANOVA, followed by the Tukey’s *post hoc* test. The data at different time points were compared by repeated measures ANOVA, followed by Bonferroni’s *post hoc* test. The cell experiment was repeated three times.

To further explore the specific mechanism of HPSC-EXO in promoting Gem resistance and tumorigenesis in pancreatic cancer, we performed differential expression analysis on the GSE32676 and GSE16515 datasets. The results showed that lncRNA UCA1 was highly expressed in pancreatic cancer samples (**Figures 3A, B**). Therefore, we speculated that HPSC-EXO may affect the biological functions of PANC-1 cells through lncRNA UCA1. The results of qRT-PCR showed that the expression of lncRNA UCA1 was significantly higher in HPSCs and HPSC-EXO than that in PSCs and PSC-EXO, respectively (**Figure 3C**). At the same time, compared with the PBS treatment, HPSC-EXO treatment increased the expression of lncRNA UCA1 in PANC-1 cells but there was no significant alteration in PANC-1 cells treated with PSC-EXO (**Figure 3D**). In addition, HPSCs were transfected with Cy3-labeled lncRNA UCA1 and then co-cultured with PANC-1 cells for 48 h. The observation results using a confocal microscope suggested that fluorescently labeled lncRNA UCA1 could be observed in PANC-1 cells (**Figure 3E**). These results indicate that HPSC-EXO transferred lncRNA UCA1 to PANC-1 cells.

Next, we set up three lncRNA UCA1 shRNA sequences. qRT-PCR results showed that sh-lncRNA UCA1-3 had the most significant effect on inhibiting the expression of lncRNA UCA1 (**Figure 3F**). Therefore, sh-lncRNA UCA1-3 was selected for subsequent experiments. qRT-PCR results showed an increase in the expression of lncRNA UCA1 in PANC-1 cells with lncRNA UCA1 silencing and a more pronounced increase was noted in sh-lncRNA UCA1-treated PANC-1 cells co-cultured with HPSC-EXO (**Figure 3G**). MTT assay results revealed reduced viability of PANC-1 cells after lncRNA UCA1 knockdown, which was restored by further co-culture with HPSC-EXO (**Figure 3H**). Moreover, the results of flow cytometry (**Figure 3I**) and TUNEL staining (**Figure 3J**) showed that sh-lncRNA UCA1 treatment alone promoted the apoptosis of PANC-1 cells, which were reversed by additional co-culture with HPSC-EXO. Western blot data also showed that the expression of cleaved caspase 3 was increased following sh-lncRNA UCA1 treatment in PANC-1 cells, while it was reduced after further co-culture with HPSC-EXO (**Figure 3K**). Furthermore, Transwell assay results indicated a decline in the migration and invasion of cells following lncRNA UCA1 knockdown while treatment with HPSC-EXO + sh-lncRNA UCA1 abolished this effect (**Figures 3L, M**). As shown in **Figure 3N**, PANC-1 cells with lncRNA UCA1 knockdown were more sensitive to Gem while further co-culture with HPSC-EXO reversed this sensitivity to a certain extent.

These results reveal that lncRNA UCA1 can be transferred to pancreatic cancer cells by HPSC-EXO, thereby promoting the malignant phenotypes of pancreatic cancer cells and their resistance to Gem.

### LncRNA UCA1 Recruits EZH2 to the Promoter of SOCS3 and Downregulates SOCS3 Expression

**Figure 4:**
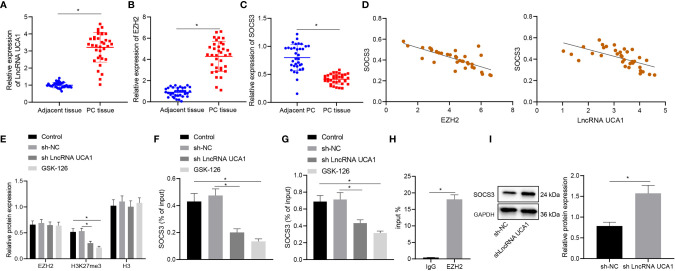
LncRNA UCA1 inhibits SOCS3 expression by recruiting EZH2 to the promoter of SOCS3 in PANC-1 cells. **(A)**, LncRNA UCA1 expression in pancreatic cancer tissues (n = 35) and adjacent normal tissues (n = 35) was detected by qRT-PCR. **(B)**, EZH2 expression in pancreatic cancer tissues (n = 35) and adjacent normal tissues (n = 35) was detected by qRT-PCR. **(C)**, SOCS3 expression in pancreatic cancer tissues (n = 35) and adjacent normal tissues (n = 35) was detected by qRT-PCR. **(D)**, Pearson’s correlation analysis between EZH2 and SOCS3 expression, SOCS3 and lncRNA UCA1 expression in pancreatic cancer tissues. **(E)**, Expression specificity of H3K27me3 in PANC-1 cells was detected by Western blot. **(F)**, Binding of EZH2 with SOCS3 promoter analyzed by ChIP. **(G)**, Binding of H3K27me3 with SOCS3 promoter was analyzed by ChIP. **(H)**, Specific binding of lncRNA UCA1 to EZH2 in PANC-1 cells was analyzed by RIP. **(I)**, Expression of SOCS3 in sh-lncRNA UCA1-treated PANC-1 cells was detected by Western blot. *p < 0.05. Measurement data were expressed as mean ± standard deviation. Paired *t* test was used for comparison between cancer tissues and adjacent normal tissues. The data between two groups were compared using the independent sample *t* test. The data comparison among multiple groups was performed using one-way ANOVA, followed by the Tukey’s *post hoc* test. Pearson’s correlation coefficient was used for correlation analysis. The cell experiment was repeated three times independently.

We then moved to illustrate the downstream mechanism of the effect of lncRNA UCA1 in pancreatic cancer. Differential expression analysis on the GSE32676 and GSE16515 datasets showed that SOCS3 was poorly expressed in pancreatic cancer samples (**Figures 3A, B**). The results of qRT-PCR showed higher expression of lncRNA UCA1 and EZH2 and lower SOCS3 expression in pancreatic cancer tissues than that in adjacent normal tissues (**Figures 4A–C**). In addition, Pearson’s correlation analysis showed that SOCS3 expression was negatively correlated with the expression of EZH2 (r = -0.75, *p* < 0.01) and lncRNA UCA1 (r = -0.73, *p* < 0.01) in pancreatic cancer tissues (**Figure 4D**).

Next, we sought to explore the regulatory relationship between EZH2 and SOCS3. In PANC-1 cells, although the total expression of EZH2 did not change, the level of H3K27me3 in response to sh-lncRNA UCA1 was reduced to the level equivalent to that of GSK-126 (EZH2 inhibitor) (**Figure 4E**). Consistently, ChIP results showed that the binding of EZH2 and H3K27me3 in the SOCS3 promoter was reduced in PANC-1 cells in response to sh-lncRNA UCA1 or GSK-126 (**Figures 4F, G**). In addition, RIP assay results showed that lncRNA UCA1 bound to EZH2 in PANC-1 cells (**Figure 4H**). Western blot results also showed that knockdown of lncRNA UCA1 in PANC-1 cells elevated the expression of SOCS3 (**Figure 4I**). These data suggest that lncRNA UCA1 is involved in EZH2 mediated epigenetic inhibition of SOCS3 in PANC-1 cells by binding to EZH2.

### LncRNA UCA1 Delivered by HPSC-EXO Promotes Gem Resistance and Tumorigenesis of Pancreatic Cancer Cells by Regulating the EZH2/SOCS3 Axis *In Vitro* and *In Vivo*

**Figure 5:**
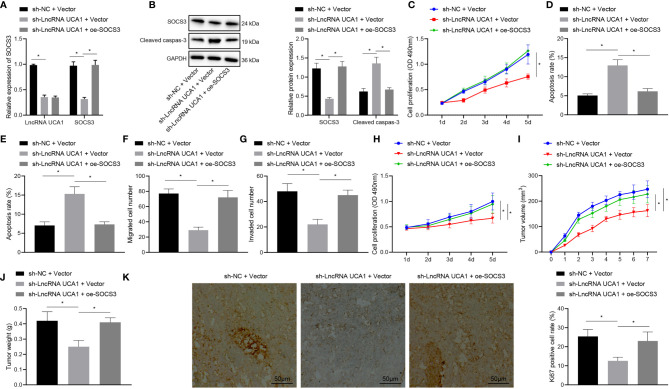
LncRNA UCA1 delivered by HPSC-EXO enhances Gem resistance and promotes tumorigenesis of pancreatic cancer by recruiting EZH2 and downregulating SOCS3 expression. **(A)**, Expression of lncRNA UCA1 and SOCS3 in PANC-1 cells treated with sh-lncRNA UCA1 or combined with oe-SOCS3 was detected by qRT-PCR. **(B)**, Expression of cleaved caspase 3 and SOCS3 in PANC-1 cells treated with sh-lncRNA UCA1 or combined with oe-SOCS3 was detected by Western blot. **(C)**, Viability of PANC-1 cells treated with sh-lncRNA UCA1 or combined with oe-SOCS3 was detected by MTT assay. **(D)**, Apoptosis of PANC-1 cells treated with sh-lncRNA UCA1 or combined with oe-SOCS3 was detected by flow cytometry. **(E)**, Apoptosis of PANC-1 cells treated with sh-lncRNA UCA1 or combined with oe-SOCS3 was detected by TUNEL staining. **(F)**, Migration of PANC-1 cells treated with sh-lncRNA UCA1 or combined with oe-SOCS3 was detected by Transwell assay. **(G)**, Invasion of PANC-1 cells treated with sh-lncRNA UCA1 or combined with oe-SOCS3 was detected by Transwell assay. **(H)**, Gem sensitivity of PANC-1 cells treated with sh-lncRNA UCA1 or combined with oe-SOCS3 analyzed by MTT method. **(I)**, Tumor volume of mice treated with sh-lncRNA UCA1 or combined with oe-SOCS3 after injection of Gem. **(J)**, Tumor weight of mice treated with sh-lncRNA UCA1 or combined with oe-SOCS3 after injection of Gem. **(K)**, The positive expression rate of Ki67 in tumor tissues of mice treated with sh-lncRNA UCA1 or combined with oe-SOCS3 after injection of Gem was detected by immunohistochemistry. *p < 0.05. Measurement data were expressed as mean ± standard deviation. The data comparison among multiple groups was performed using one-way ANOVA, followed by the Tukey’s *post hoc* test. The data at different time points were compared by repeated measures ANOVA, followed by Bonferroni’s *post hoc* test. The cell experiment was repeated three times. n = 8 for mice following each treatment.

Finally, we proceeded to determine whether HPSC-EXO affects the chemotherapy resistance of pancreatic cancer through the lncRNA UCA1/EZH2/SOCS3 axis. The results of qRT-PCR and Western blot showed that the expression of lncRNA UCA1 and SOCS3 was reduced in PANC-1 cells following lncRNA UCA1 knockdown while further SOCS3 overexpression enhanced the expression of SOCS3 (**Figures 5A, B**).

Moreover, the results of MTT assay (**Figure 5C**) revealed a reduction in the viability of PANC-1 cells upon knockdown of lncRNA UCA1, while an enhancement was noted in the presence of both lncRNA UCA1 knockdown and SOCS3 overexpression. As depicted in **Figures 5B, D, E**, lncRNA UCA1 knockdown augmented cell apoptosis and increased the expression of cleaved caspase 3, the effect of which was abolished by further SOCS3 overexpression. Furthermore, the results of Transwell assay exhibited that lncRNA UCA1 knockdown resulted in inhibition in the migration and invasion of PANC-1 cells, which was negated by additional SOCS3 overexpression (**Figures 5F, G**). MTT assay data (**Figure 5H**) showed that PANC-1 cells treated with sh-lncRNA UCA1 + oe-SOCS3 had reduced sensitivity to Gem than those with sh-lncRNA UCA1 alone.

The results of *in vivo* experiments (**Figures 5I–K**) further showed that treatment with Gem reduced the tumor volume and weight of mice with lncRNA UCA1 knockdown as well as decreasing the positive expression rate of Ki67 in the tumor tissues. However, opposite results were noted in the presence of sh-lncRNA UCA1 + oe-SOCS3. The above results indicate that lncRNA UCA1 regulates the EZH2/SOCS axis to promote the Gem resistance and tumorigenesis of pancreatic cancer cells.

## Discussion

As a typical feature of most tumors, especially solid tumors, hypoxia microenvironment induces adaptive regulation of tumor and stromal cells, thus promoting the development and invasiveness of tumors (17). Tumor cells have been shown to release more exosomes in hypoxia microenvironment, which are involved in angiogenesis regulation (18). In the present study, we found that the hypoxic tumor microenvironment could induce activation of PSCs and enhance the secretion of exosomes from PSCs. The high expression of exosomal lncRNA UCA1 promoted the malignant phenotypes, and augmented the Gem resistance of pancreatic cancer.

Notably, the expression of lncRNA UCA1 is dysregulated in many malignant tumors, which affects tumor occurrence and development. Some studies have also reported that lncRNA UCA1 is highly expressed in pancreatic cancer tissues and plays a carcinogenic role in tumor proliferation and metastasis (19, 20). In addition, lncRNA UCA1 expression was found not only to be increased in hypoxic pancreatic cancer cells, but also enriched in exosomes derived from hypoxic pancreatic cancer cells, and it was established that lncRNA UCA1 knockout restricted angiogenesis and tumor growth (7). These results suggested that hypoxic pancreatic cancer cell-derived exosome-mediated angiogenesis was dependent on lncRNA UCA1.

It is well acknowledged that lncRNAs function *via* various mechanisms, which are mainly based on the cellular location. We also showed that knockdown of lncRNA UCA1 inhibited tumorigenesis in mice xenografting with pancreatic cancer cells. EZH2 overexpression occurred in pancreatic cancer and it was positively correlated with lncRNA UCA1 expression. Previous evidence showed that lncRNA UCA1 was up-regulated in hepatocellular carcinoma, and EZH2 showed a negative correlation with lncRNA UCA1 level (12). EZH2 is a lysine methyltransferase, which can synergize with lncRNA UCA1. LncRNA UCA1 was up-regulated in non-small-cell lung cancer tissues and gefitinib resistant cells, indicating that lncRNA UCA1 played a special role in gefitinib resistance, and lncRNA UCA1 knockout can increase the sensitivity to gefitinib by inhibiting cell proliferative capacities by interacting with EZH2 to epigenetically reduce CDKN1A expression (21). miR-124 delivered by bone marrow mesenchymal stromal cell-derived exosomes can directly inhibit the expression of EZH2, and consequently suppress the malignant phenotypes of pancreatic tumor cells, and sensitizes pancreatic cancer cells to chemotherapy *in vitro* and *in vivo* (22). In addition, tumor-derived exosomal LINC01133 can interact with EZH2 and then promotes H3K27 trimethylation, contributing to pancreatic ductal adenocarcinoma tumor growth and epithelial-mesenchymal transition *in vivo* (23).

Further, we observed that SOCS3 underexpression occurred in pancreatic cancer and it was negatively correlated with EZH2. In addition, lncRNA UCA1 was involved in EZH2 mediated epigenetic inhibition of SOCS3 in PANC-1 cells by binding to EZH2. It was previously found that the expression of SOCS3 in hepatocellular carcinoma tissues and cell lines was negatively correlated with EZH2 and depended on its promoter methylation status through TCGA hepatocellular carcinoma data analysis. The promoter recruitment of SOCS3 gene by CTCF dependent EZH2 may be involved in the epigenetic silencing of SOCS3 and the regulation of its gene expression (13). It was shown in *in vivo* and *in vitro* experiments of a previous study, upregulated GAS5 promoted the expression of SOCS3, thus inhibiting the growth, metastasis, and Gem resistance of pancreatic cancer (14). Corroborating previous documentation, our results confirmed through gain- and loss- of function assays that lncRNA UCA1 inhibited the expression of SOCS3, thereby enhancing the malignant phenotypes, tumorigenesis and Gem resistance of pancreatic cancer cells.

## Conclusion

**Figure 6:**
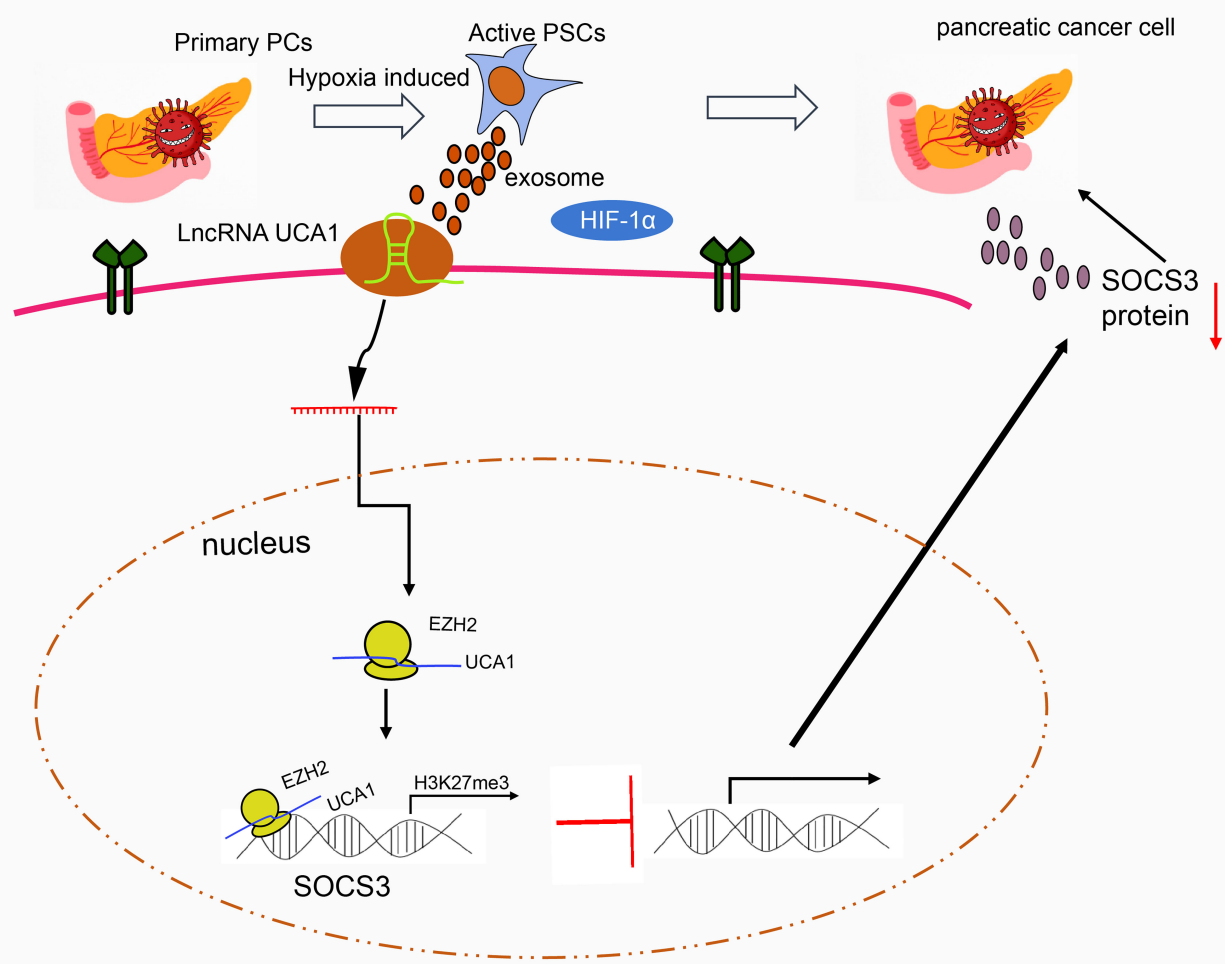
The molecular mechanism of lncRNA UCA1 loaded by HPSC-EXO in pancreatic cancer. Under hypoxic conditions, the exosomes secreted by PSCs transfer lncRNA UCA1 to pancreatic cancer cells, where lncRNA UCA1 recruits EZH2 to the SOCS3 promoter to increase the histone methylation modification and inhibit the transcription expression of SOCS3. By this mechanism, the resistance of pancreatic cancer cells to Gem is enhanced.

In conclusion, the exosomes secreted by hypoxia-activated PSCs could deliver lncRNA UCA1 to pancreatic cancer cells. By recruiting EZH2, lncRNA UCA1 regulates the methylation of SOCS3 protein, reduces the expression of SOCS3, and promotes the Gem resistance of pancreatic cancer (**Figure 6**). Thus, our study provides interesting targets for pancreatic cancer prevention and treatment. Future studies are warranted to understand the mechanistic link between our findings and potential association with tumor reactive oxygen species, and the ensuing instability of tumor genome and formation of new blood vessels.

## Data Availability Statement

The original contributions presented in the study are included in the article/**Supplementary Material**. Further inquiries can be directed to the corresponding author.

## Ethics Statement

The studies involving human participants were reviewed and approved by the Ethics Committee of Shengjing Hospital of China Medical University. The patients/participants provided their written informed consent to participate in this study. The animal study was reviewed and approved by the Animal Ethics Committee of Shengjing Hospital of China Medical University.

## Author Contributions 

YC and ZL designed the study and drafted the paper. HX and ZL were involved in data collection. YC and HX performed the statistical analysis and preparation of figures. All authors contributed to the article and approved the submitted version.

## Funding

This study was supported by General projects of the National Natural Science Foundation of China (8187071004) and the 345 Talent Project.

## Conflict of Interest

The authors declare that the research was conducted in the absence of any commercial or financial relationships that could be construed as a potential conflict of interest.

## Publisher’s Note

All claims expressed in this article are solely those of the authors and do not necessarily represent those of their affiliated organizations, or those of the publisher, the editors and the reviewers. Any product that may be evaluated in this article, or claim that may be made by its manufacturer, is not guaranteed or endorsed by the publisher.

## Supplementary Material

Supplementary Figure 1 Gem resistance assay. **(A)**, Dose-response curve of Gem in the parental and GemMIA-R3 cell lines. Data represent the mean ± standard error of at least three independent experiments. **(B)**, Cell growth inhibition data (GI50) of the parental and three Gem-resistant MIA PaCa-2 cell lines. Unit: nM. The cell experiment was repeated three times.
