# Supplementary figures and images for: Exosomal lncRNA UCA1 Derived From Pancreatic Stellate Cells Promotes Gemcitabine Resistance in Pancreatic Cancer via the SOCS3/EZH2 Axis
Source: Front Oncol. 2021 Nov 19;11:671082. doi: 10.3389/fonc.2021.671082 (PMC8640181; doi:10.3389/fonc.2021.671082)

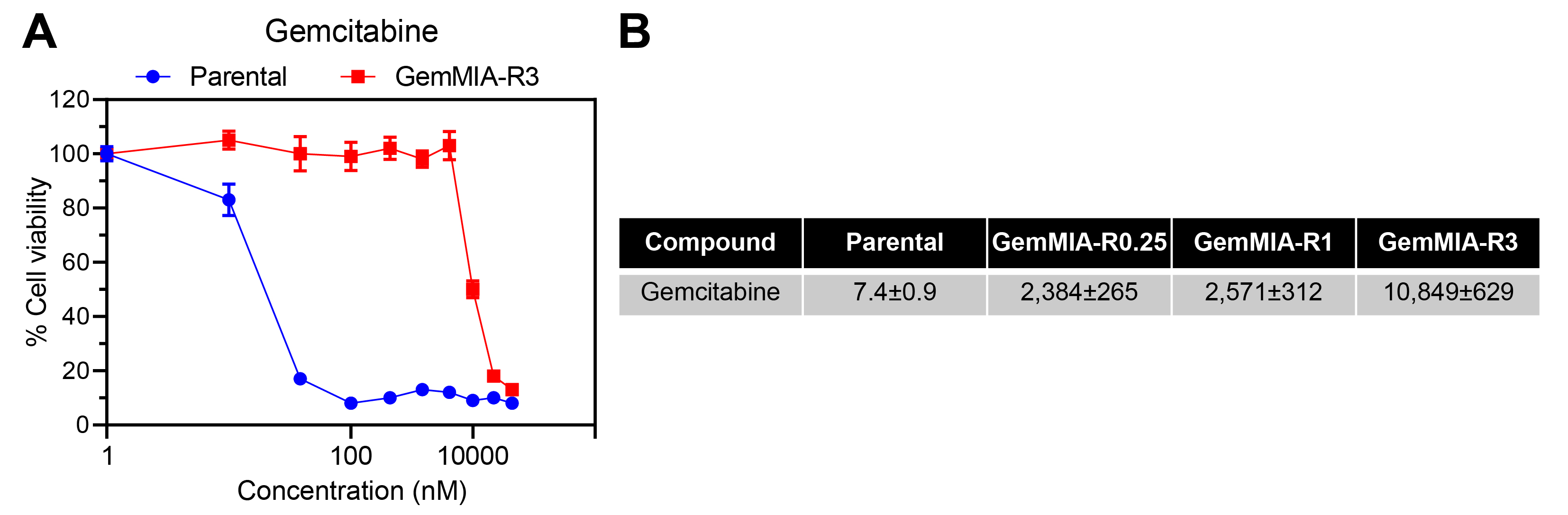

Supplement: Supplementary Figure 1 — Gem resistance assay. (A), Dose-response curve of Gem in the parental and GemMIA-R3 cell lines. Data represent the mean ± standard error of at least three independent experiments. (B), Cell growth inhibition data (GI50) of the parental and three Gem-resistant MIA PaCa-2 cell lines. Unit: nM. The cell experiment was repeated three times. [file Image_1.jpeg]
