# Supplementary material for: Exosomal lncRNA UCA1 Derived From Pancreatic Stellate Cells Promotes Gemcitabine Resistance in Pancreatic Cancer via the SOCS3/EZH2 Axis
Source: Front Oncol. 2021 Nov 19;11:671082. doi: 10.3389/fonc.2021.671082 (PMC8640181; doi:10.3389/fonc.2021.671082)
Supplement: Supplementary file 2 [file Table_1.docx]

**Supplementary Table 1** Cell grouping

| Group | Treatment |
| --- | --- |
| PSC | PSCs cultured under normal conditions |
| HPSC | PSCs cultured under hypoxia |
| PBS | PSCs cultured under normal conditions treated with PBS |
| PSC-EXO | PSC-EXO co-cultured with pancreatic cancer cells |
| HPSC-EXO | HPSC-EXO co-cultured with pancreatic cancer cells |
| sh-NC | Pancreatic cancer cells transfected with sh-NC |
| sh-lncRNA UCA1 | Pancreatic cancer cells transfected with sh-lncRNA UCA1 |
| HPSC-EXO + sh-NC | HPSC-EXO co-cultured with pancreatic cancer cells transfected with sh-NC |
| HPSC-EXO + sh-lncRNA UCA1 | HPSC-EXO co-cultured with pancreatic cancer cells transfected with sh-lncRNA UCA1 |
| sh-NC + Vector | Pancreatic cancer cells transfected with sh-NC and Vector |
| sh-lncRNA UCA1 + Vector | Pancreatic cancer cells transfected with sh-lncRNA UCA1 and Vector |
| sh-lncRNA UCA1 + oe-SOCS3 | Pancreatic cancer cells transfected with sh-lncRNA UCA1 and oe-SOCS3 |

**Note:** PSC, pancreatic stellate cell; HPSC, hypoxic pancreatic stellate cell; PSC-EXO, exosomes derived from PSCs; HPSC-EXO, exosomes derived from HPSCs; sh-, short hairpin RNA; oe- overexpression; NC, negative control
