# Supplementary material for: Exosomal lncRNA UCA1 Derived From Pancreatic Stellate Cells Promotes Gemcitabine Resistance in Pancreatic Cancer via the SOCS3/EZH2 Axis
Source: Front Oncol. 2021 Nov 19;11:671082. doi: 10.3389/fonc.2021.671082 (PMC8640181; doi:10.3389/fonc.2021.671082)
Supplement: Supplementary file 3 [file Table_2.docx]

**Supplementary Table 2** Primer sequences for qRT-PCR

| Genes | Primer sequences (5’-3’) |
| --- | --- |
| LncRNA UCA1 | Forward: CCCTACCCCAGTAATCCCCA |
|  | Reverse: AGACTGCCTTTGGGTTGAGG |
| HIF-1α | Forward: AGAGGTTGAGGGACGGAGAT |
|  | Reverse: GCACCAAGCAGGTCATAGGT |
| EZH2 | Forward: GAAGCAGGGACTGAAACGG |
|  | Reverse: ATTGAGGCTTCAGCACCACT |
| SOCS3 | Forward: CACACCGGACCAACCAGC |
|  | Reverse: CTGTCGCGGATCAGAAAGGT |
| GAPDH | Forward: GCACCGTCAAGGCTGAGAAC |
|  | Reverse: TGGTGAAGACGCCAGTGGA |
