# Supplementary material for: Exosomal lncRNA UCA1 Derived From Pancreatic Stellate Cells Promotes Gemcitabine Resistance in Pancreatic Cancer via the SOCS3/EZH2 Axis
Source: Front Oncol. 2021 Nov 19;11:671082. doi: 10.3389/fonc.2021.671082 (PMC8640181; doi:10.3389/fonc.2021.671082)
Supplement: Supplementary file 4 [file Table_3.docx]

**Supplementary Table 3** Antibody information for western blot

| Antibodies | Information |
| --- | --- |
| HIF-1α | 1/1000, ab179483, Abcam Inc., Cambridge, UK |
| Cleaved caspase 3 | 1/500, ab32042, Abcam Inc., Cambridge, UK |
| EZH2 | 1:500, 36-6300, Thermo Fisher Scientific Inc., Waltham, MA, USA |
| H3K27me3 | 1/100, ab6002, Abcam Inc., Cambridge, UK |
| CD9 | 1/2000, ab92726, Abcam Inc., Cambridge, UK |
| CD63 | 1/1000, ab134045, Abcam Inc., Cambridge, UK |
| IgG | 1:5000, ab6721, Abcam Inc., Cambridge, UK |
| H3 | 1:3000, ab1791, Abcam Inc., Cambridge, UK |
| GAPDH | 1/2000, ab8245, Abcam Inc., Cambridge, UK |
