# Supplementary material for: Exosomal lncRNA UCA1 Derived From Pancreatic Stellate Cells Promotes Gemcitabine Resistance in Pancreatic Cancer via the SOCS3/EZH2 Axis
Source: Front Oncol. 2021 Nov 19;11:671082. doi: 10.3389/fonc.2021.671082 (PMC8640181; doi:10.3389/fonc.2021.671082)
Supplement: Supplementary file 5 [file Table_4.docx]

**Supplementary Table 4** Primer sequences for ChIP

| Gene | Sequences (5’-3’) |
| --- | --- |
| SOCS3 | Forward: CGCTTCGGGACTAGGTAGGA |
|  | Reverse: AGAAACCGGGAAAAGCTCCC |
